# N-1 Perfusion Platform Development Using a Capacitance Probe for Biomanufacturing

**DOI:** 10.3390/bioengineering9040128

**Published:** 2022-03-22

**Authors:** Emily S. C. Rittershaus, Matthew S. Rehmann, Jianlin Xu, Qin He, Charles Hill, Jeffrey Swanberg, Michael C. Borys, Zheng-Jian Li, Anurag Khetan

**Affiliations:** Biologics Development, Global Product Development and Supply, Bristol Myers Squibb Company, Devens, MA 01434, USA; emily.rittershaus@bms.com (E.S.C.R.); matthew.s.rehmann@gmail.com (M.S.R.); qin.he@bms.com (Q.H.); chill@repligen.com (C.H.); jeffrey.swanberg@bms.com (J.S.); michael.borys@bms.com (M.C.B.); zli@horizontherapeutics.com (Z.-J.L.); anurag.khetan@bms.com (A.K.)

**Keywords:** perfusion N-1, process intensification, capacitance, process analytical technologies (PAT), cell-specific perfusion rate (CSPR), platform, scale-up, in-line probe, monoclonal antibody (mAb), Chinese hamster ovary (CHO)

## Abstract

Fed-batch process intensification with a significantly shorter culture duration or higher titer for monoclonal antibody (mAb) production by Chinese hamster ovary (CHO) cells can be achieved by implementing perfusion operation at the N-1 stage for biomanufacturing. N-1 perfusion seed with much higher final viable cell density (VCD) than a conventional N-1 batch seed can be used to significantly increase the inoculation VCD for the subsequent fed-batch production (referred as N stage), which results in a shorter cell growth phase, higher peak VCD, or higher titer. In this report, we incorporated a process analytical technology (PAT) tool into our N-1 perfusion platform, using an in-line capacitance probe to automatically adjust the perfusion rate based on real-time VCD measurements. The capacitance measurements correlated linearly with the offline VCD at all cell densities tested (i.e., up to 130 × 10^6^ cells/mL). Online control of the perfusion rate via the cell-specific perfusion rate (CSPR) decreased media usage by approximately 25% when compared with a platform volume-specific perfusion rate approach and did not lead to any detrimental effects on cell growth. This PAT tool was applied to six mAbs, and a platform CSPR of 0.04 nL/cell/day was selected, which enabled rapid growth and maintenance of high viabilities for four of six cell lines. In addition, small-scale capacitance data were used in the scaling-up of N-1 perfusion processes in the pilot plant and in the GMP manufacturing suite. Implementing a platform approach based on capacitance measurements to control perfusion rates led to efficient process development of perfusion N-1 for supporting high-density CHO cell cultures for the fed-batch process intensification.

## 1. Introduction

Despite much interest from the biopharmaceutical industry in continuous upstream manufacturing processes, fed-batch processes remain the standard mode of production for stable proteins such as monoclonal antibodies (mAbs). A conventional fed-batch process is usually inoculated with an initial viable cell density (VCD) of <1 × 10^6^ cells/mL. The volumetric productivity and manufacturing throughput of a fed-batch process can be improved by inoculating the production bioreactor at a higher initial VCD (e.g., 2–8 × 10^6^ cells/mL), thus shortening the initial growth phase and total culture duration [1,2,3,4]. Furthermore, an even higher initial VCD (e.g., 10–20 × 10^6^ cells/mL) can double fed-batch cell culture titers [5,6], which are comparable to the high end of the best titers reported in the current literature [7].

Inoculation of the production bioreactor at a higher initial VCD requires the N-1 to achieve a higher final VCD (e.g., 14–30 × 10^6^ cells/mL), which can be achieved through intensification of the N-1 with non-perfusion [8] or perfusion strategies [1,2]. However, only perfusion N-1 has demonstrated the feasibility of achieving final VCDs of greater than 60 to 100 × 10^6^ cells/mL [9], enabling inoculation VCDs in the production bioreactor of greater than 10 × 10^6^ viable cells/mL [5,6]. Because the fed-batch process intensification can achieve a great titer improvement or shortened culture duration by N-1 perfusion while remaining cost effective compared to traditional fed batch (overall media USD/g mAb) [10], the N-1 perfusion strategy has been widely used for mAb production in the cell culture industry [11,12].

One common method of controlling perfusion rate is to perfuse a fixed volume that changes at a pre-defined rate with time, typically normalized to the working volume of the bioreactor and reported in vessel volumes per day (VVD). While this volume-specific exchange rate strategy is relatively straightforward in implementation, this strategy tends to perfuse more media than the cells require to maintain in a healthy state to avoid the alternative condition where the perfused volume is too low, leaving the cells deficient in required nutrients [12]. The perfusion of excess media is problematic during scale-up of perfusion processes, where preparation and storage of large media volumes is challenging and is often limited by the size of the equipment in the manufacturing facility [13]. An elegant solution to this problem is to perfuse on a cell-specific basis rather than a volume-specific basis [14]. The cell-specific perfusion rate (CSPR) describes the required amount of fresh media supplied to the culture on a per-cell basis and the equivalent amount of spent media perfused out through the cell retention device [15].

A perfusion strategy based on the CSPR conceptually can supply the cell culture with the precise amount of media required to support cell growth at all VCDs, controlling cost while enabling improved productivity associated with these perfusion processes. However, the timescale for typical VCD measurements (i.e., once per day by trypan blue exclusion) is too infrequent compared with the timescale of cell growth, especially at high cell densities where the exponentially expanding cell culture can grow significantly within hours. A process analytical technology (PAT) tool, in contrast, enables real-time or near real-time measurement of critical process parameters such as VCD [16]. The most common options for using PAT to measure the VCD of CHO cell cultures in real time include an autosampler connected to a cell counter [17,18], a capacitance probe [19,20,21,22], and a Raman probe [23,24,25,26], but the capacitance probe is of particular interest in perfusion cell culture applications for several reasons. Importantly, capacitance is a relatively direct measurement of VCD. The basic principle of capacitance technology is as follows: Intact cells act as small capacitors in the presence of an electric field, thus the capacitance of the cell culture solution is proportional to viable cell volume [27]. Capacitance probe technology has existed for over forty years, and many of the challenges previously reported, such as interference from gassing and agitation, have now been overcome with improvements in data collection and analysis [27,28]. In addition, the capacitance probe offers non-destructive, rapid, real-time measurements, can accurately measure VCD up to high cell densities [9], and is a GMP-compatible technology, with use already reported in pharmaceutical manufacturing applications [29]. Furthermore, current capacitance instruments can be integrated with bioreactor control systems such as DeltaV and used as a control method for automatically adjusting the perfusion rate based on the VCD [30]. 

We and others have reported the use of an in-line capacitance probe in the N-1 perfusion bioreactor, which is capable of accurately providing and controlling perfusion rates based on real-time VCD measurements [3,9]. However, to the best of our knowledge, there are no current reports detailing the development of capacitance sensors for the manufacture of many molecules as part of N-1 perfusion in an intensified cell culture platform. A manufacturing platform is a framework for developing a production process for a family of related molecules, e.g., monoclonal antibodies using CHO cell expression systems. Incorporation of a technology into a platform process goes beyond demonstrating the successful proof-of-concept of a technology and requires demonstrating applicability of the conclusions to many CHO clones to build confidence that the process will work for future molecules. In addition, an effective cell culture platform should define a starting set of cell culture conditions that work reasonably well for most products in that family and include a development workflow with which a process can be efficiently optimized for improved performance or robustness through process development.

In previous reports, we described the evolution of our manufacturing platform from conventional to intensified processes [5] and demonstrated that increasing inoculation density, coupled with media optimization, led to titer improvement at the production bioreactor stage [6]. This report builds on the previous two with a focus on the N-1 stage optimization that enabled the increased inoculation density implemented at the production stage. Specifically, we have incorporated capacitance-controlled N-1 perfusion into our cell culture platform process, and in this report, we detail considerations that went into that decision. Specifically, we demonstrate that the capacitance probe provides a good estimation of VCD for many (6) different mAb molecules and enables a starting point for process development with decreased media usage when compared with volumetric-based strategies. We demonstrate that the capacitance-controlled perfusion can be rapidly optimized within the span of a few experiments to arrive at an effective N-1 perfusion process and successful scalability of the perfusion processes developed with this strategy. Finally, we demonstrate that if required for manufacturing facility fit, the capacitance probe can be engineered out of the process by converting back to a volumetric-based perfusion strategy, enabling flexibility for use of these processes in multiple manufacturing facilities.

## 2. Materials and Methods

### 2.1. Cell Lines, Media, and Seed Train

Internally developed CHO GS cell lines were used to produce proprietary mAbs 1 to 6. Cell lines expressing mAbs 1 to 6 used the same proprietary chemically defined expansion/perfusion medium with either 6 or 8 g/L of glucose (Table 1) and either 6.25 or 25 µM MSX [31]. Cells were cultured in 250 mL, 500 mL, 1 L, 3 L baffled shake flasks (Corning) at 36.5 °C, 150 rpm (110 rpm for 3 L baffled flasks), and 5% CO_2_ in a humidified incubator with a shaking diameter of 25 mm (Climo-Shaker ISF1-X, Kuhner, Basel, Switzerland). Shake flasks were seeded at approximately 0.3–0.7 × 10^6^ viable cells/mL and were subcultured every 3 to 4 days when reaching an approximate VCD range of 3–10 × 10^6^ cells/mL and minimum of 90% viability prior to the inoculation of an N-2 perfusion cell bag (Cytiva, Marlborough, MA, USA) or directly to the N-1 perfusion bioreactor. Operation of perfusion cell bags was as described in Yongky et al., 2019 [8].

### 2.2. Analytical Methods

Bioreactors were typically sampled daily. Offline VCD and viability at all scales were measured using a ViCell XR (Beckman Coulter, Loveland, CO, USA). Metabolites (ammonia, glucose, glutamate, glutamine, lactate) for the lab and pilot-scale runs (i.e., at 5 L and 200 L scale) were measured using a Cedex Bio HT (Roche, Basel, Switzerland), and offline pH, CO_2_ (mmHg), air saturation were measured by pHOx (Nova Biomedical, Waltham, MA, USA). Metabolites and dissolved gases for the manufacturing-scale bioreactors (i.e., 500 L scale) were measured using the BioProfile FLEX2 (Nova Biomedical). 

### 2.3. Laboratory Scale N-1 Perfusion Bioreactor with Online Capacitance Control

Laboratory scale 5 L benchtop glass stirred bioreactors (Sartorius UniVessel, Göttingen, Germany) with Finesse DeltaV controllers were equipped with Alternating Tangential Flow (ATF) XCell ATF2 perfusion filters (Repligen, Boston, MA, USA) and Incyte capacitance probes (Hamilton, Franklin, MA, USA). Bioreactors were equipped with CO_2_ sparging to control pH below an upper deadband of 7.4. The dissolved oxygen set point was 40%; bioreactors used both a drilled hole sparger and microsparger with O_2_ sparging to maintain the set point. Agitation was set to 260 to 320 rpm depending on the cell density, as higher cell densities often required a higher agitation rate to maintain 40% O_2_ saturation. Antifoam C (SAFC, Sigma Millipore; 3000 ppm) was used as needed to control foam levels in the bioreactors. The working volume of the reactor was set between 3.0 and 4.0 L. Durations of perfusion N-1 processes varied between 5 and 7 days depending on the mAb and the desired final VCD. 

Perfusion was initiated one day after inoculation using the XCell ATF2 perfusion filter system from Repligen. ATF2 filters were polyethersulfone fiber chemistry with a pore size of 0.2 µm, which was recently reported to be optimal for minimizing ATF column clogging [32]. Perfusion was either run at a volume specific exchange rate or using online measurements collected with a capacitance probe (Hamilton) to control at a fixed CSPR between 0.02 and 0.12 nL/cell/day; CSPR values used for each experiment are described in the Results section. Perfusion rates were calculated as follows:

$$\begin{array}{l} \mathrm{Perfusion}\;\mathrm{rate}\;\left(\frac{\mathrm{mL}}{\min}\right)\\ =\frac{\mathrm{CSPR}\;\times \;\mathrm{Capacitance}\;\mathrm{correction}\;\mathrm{factor}\times \;\mathrm{Capacitance}\;\mathrm{probe}\;\mathrm{value}\;\times \;\mathrm{Working}\;\mathrm{volume}\;\left(\mathrm{mL}\right)}{1440\;\left(\mathrm{minutes}\right)} \end{array}$$

**Figure 1:**
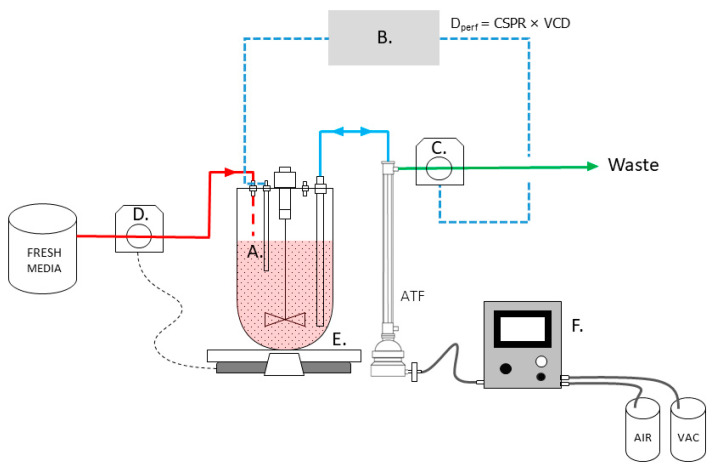
Depiction of online capacitance probe perfusion N-1 control strategy. (A) Capacitance probe. (B) Capacitance/DeltaV controller, perfusion rate calculation, D_perf_, is based on CSPR and VCD. (C) Perfusion pump, drives perfusion rate, D_perf_. (D) Media inlet pump, controlled by bioreactor scale. (E) Bioreactor scale. (F) ATF controller, controls ATF cycle rate.

The ATF exchange rate was 0.8 to 0.9 L/min with a residence time of 60 s. Alternating pressurization and exhaustion cycles were supplied to the ATF by a C24 ATF controller (Repligen). A fixed working volume was maintained by applying the perfusion rate (either volume specific or through capacitance probe control) in mL/minute to the perfusate pump and pumping fresh media into the bioreactor as needed to maintain a set point working volume using the reactor scale weight (Sartorius, Göttingen, Germany) (Figure 1). The volume-specific exchange rate, referring to the offline, predefined volume vessels per day (VVD) perfusion strategy, is aligned with the terminology used in Stepper et al. (2020) [33].

The online capacitance measurement, reported as permittivity (pF/cm), was correlated to the offline VCD (ViCell) measurement via a constant referred to herein as the capacitance correction factor. This constant was determined for each individual mAb using the slope of the best fit line when plotting the permittivity (pF/cm) against VCD (×10^6^ cells/mL). The capacitance measurement mode was set to 1 MHz.

### 2.4. N-1 Scale Up Equipment

For certain mAbs, the bench-scale process was scaled up to 200 L in the pilot plant and/or 500 L in the GMP manufacturing facility. Both the 200 and the 500 L N-1 bioreactors were single-use XCellerex bags (Cytiva). The 200 and 500 L bioreactors utilized the XCell ATF6 and were run at an ATF exchange rate of 17.3 L/min. Scale-up agitations were determined based on empirical evidence and past project experience. Respectively, 200 and 500 L agitation were approximately 120 and 110 rpm. Gassing parameters were matched based on the airflow rate per unit volume (VVM) at the 5 L scale. The working volume was set between 80 to 85 L for the 200 L reactor, and 190 L to 215 L for the 500 L reactors, respectively. The same capacitance probes (Hamilton Incyte) were used across all scales in this study.

## 3. Results

### 3.1. Online to Offline Cell Density Correlation

**Figure 2:**
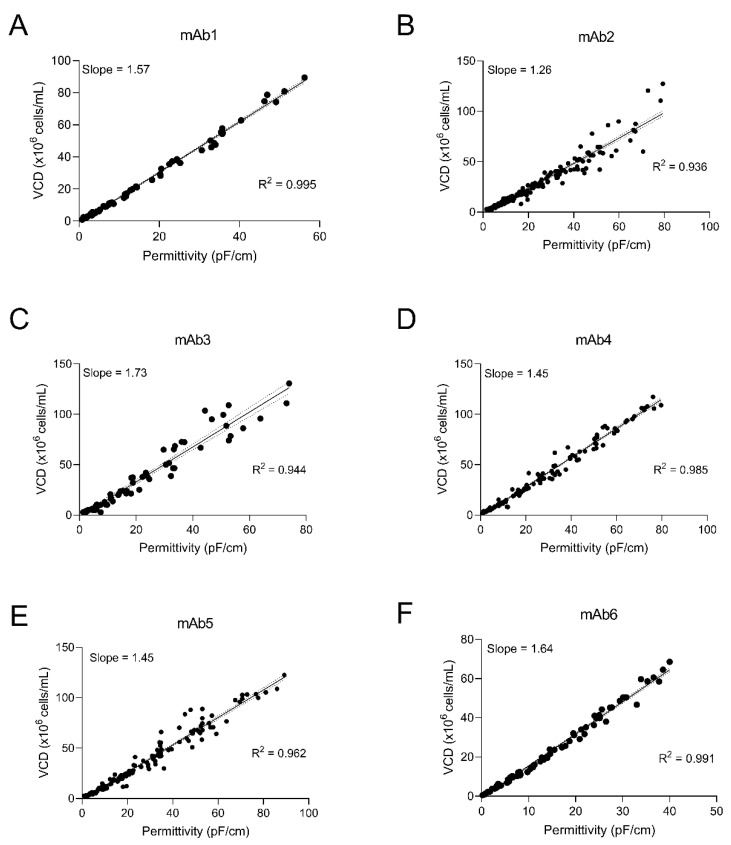
Correlation of measured permittivity with offline measured VCD. Individual measurements for mAbs 1–6 (**A**–**F**) aggregated from 5 L runs are plotted with simple linear regression and 95% confidence intervals. Data points were aggregated from the following number of 5 L runs for mAbs 1–6 are as follows: 8 (**A**), 25 (**B**), 11 (**C**), 22 (**D**), 26 (**E**), 14 (**F**), respectively.

The capacitance probe was implemented during N-1 perfusion development of six different CHO clones expressing different recombinant antibody products. To utilize the online capacitance measurement to dynamically control the perfusion rate, we first confirmed that the relationship between VCD and permittivity was linear over the VCD ranges of interest for these perfusion cultures. Capacitance measurements were captured at the same time as offline sampling, and the permittivity values were plotted against the corresponding offline VCD measurements (Figure 2). Since the cell culture remains in the exponential growth phase during the N-1 process, the online capacitance reading and the offline VCD exhibited a linear relationship from relatively low VCDs (i.e., 1.2 to 4.2 × 10^6^ viable cells/mL) to high VCDs (i.e., 80 to 130 × 10^6^ viable cells/mL). The R^2^ values were high (from 0.936 to 0.995), confirming that the online permittivity value multiplied by the capacitance probe correction factor results in a reliable prediction of VCD for all six clones in these exponentially growing perfusion cultures in the VCD ranges tested (Figure 2). 

We determined the capacitance probe correction factor that relates the online permittivity readings to the offline VCD. Each clone had a unique slope which varied between 1.26 and 1.73 (Figure 2A–F). The slope was used as the capacitance probe correction factor to calculate the perfusion rate as a function of the CSPR.

### 3.2. Cell-Specific Perfusion Control by a Capacitance Probe Decreases Media Usage in Perfusion N-1 Bioreactors

One major challenge in scale-up of N-1 perfusion processes is managing the large media volumes associated with running perfusion processes at large scale. Thus, it is desirable to develop a process that supports growth of a high cell density culture with high viability while minimizing the amount of media required for perfusion. A side-by-side comparison of N-1 perfusion processes using mAb1 and mAb5 with either a volume-specific exchange rate or online capacitance probe control of the perfusion rate was carried out to compare media usage between the two strategies. 

**Figure 3:**
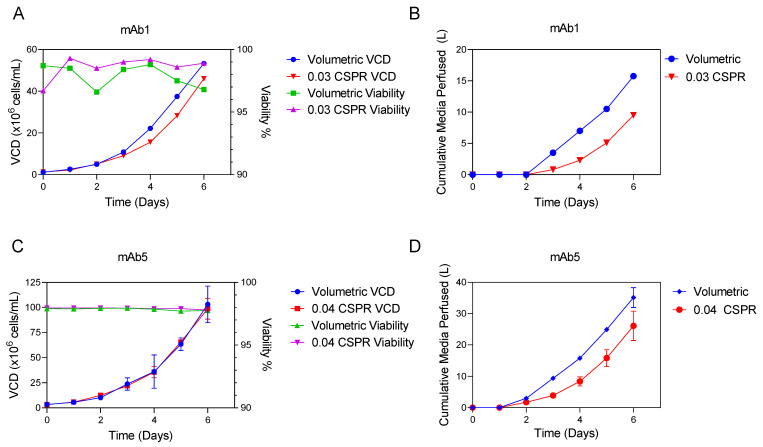
VCD and viability and cumulative media perfused for volumetric (volume-specific exchange rate) and online CSPR controlled N-1 for mAb1 and mAb5. Performance comparison for two mAbs using either a volume specific exchange rate or online controlled CSPR. For mAb1, VCD and viability are plotted in (**A**) and cumulative media is plotted in (**B**). For mAb 5, VCD and viability are plotted in (**C**) and cumulative media is plotted in (**D**). For mAb1, n = 1; for mAb5, n = 3. In panel (**C**,**D**), data are plotted as average ± standard deviation.

mAb1 was inoculated at 1.2 × 106 cells/mL in two 5 L bioreactors, each of which was configured with an ATF2, with the goal of achieving a final VCD of 45 to 50 × 106 cells/mL. Based on internal experience with this perfusion medium, we anticipated that the perfusion rate would need to be approximately 1.5 VVD to ensure a healthy cell culture at this target final VCD. Therefore, the volume-specific exchange rate condition was configured to perfuse 1 VVD on days 2 to 5 and was increased to 1.5 VVD from day 5 to 6. The second bioreactor was run at 0.03 nL/cell/day CSPR from day 2 to 6 and the VCD calculated from the capacitance measurement was used in the Finesse DeltaV system for perfusion pump control. As intended, the media perfused into the bioreactor increased in real-time as the VCD increased. On day 6 of the process, both the volume-specific exchange rate and online capacitance probe-controlled experimental groups achieved similar final VCD and viability; for mAb1, the bioreactor controlled with a volume-specific exchange rate grew to 53.3 × 106 cells/mL with 96.8% viability, and the bioreactor controlled with the online CSPR grew to 46.0 × 106 cells/mL with 98.9% viability (Figure 3B). Although the VCD with the online CSPR control was approximately 15% lower than the volume-specific exchange rate control, the condition controlled with the online CSPR perfused 39% less media for mAb1. Taking final VCD into consideration, the online CSPR control saved 24% media.

For mAb5, N-1 reactors were inoculated at 3.3 × 106 cells/mL and perfusion was initiated one day post-inoculation. For this mAb, the target final VCD in the N-1 was 95 to 100 × 106 cells/mL. Based on internal experience with this media type, we anticipated that the required final flow rate to support this cell density and maintain high viability in our stepwise gradient needed to be approximately 4 VVD. Therefore, the perfusion rate in the volume-specific exchange rate condition used a stepwise gradient of 1 VVD from day 1 to 2, 2 VVD from day 2 to 3, 3 VVD from day 3 to 4, and 4 VVD from day 5 to 6. The online-controlled perfusion condition using the capacitance probe was run at 0.04 nL/cell/day CSPR. For mAb5, the bioreactor controlled with the volume-specific exchange rate grew to 103.1 × 106 cells/mL with 97.0% viability, and the bioreactor controlled with the online CSPR grew to 98.7 × 106 cells/mL with 97.5% viability (Figure 3D). Consistent with the findings for mAb1, both conditions achieved the target final VCD while maintaining high cell viabilities, but the CSPR-mediated perfusion control used 26% less media for mAb5 (Figure 3A,C). 

This experiment shows the promise of the CSPR-based perfusion control strategy for use in a perfusion platform. One feature of a platform process is that it aims to provide a good starting point for process development. For both mAb1 and mAb5, the initial perfusion rate design with the CSPR-based strategy demonstrated adequate cell growth with less total media perfused; thus, the CSPR-based approach allowed for development of both mAbs to begin at a more optimized process condition. The offline volume-specific exchange rate strategy would have required additional development effort to achieve a reduction in media use to levels comparable to the CSPR-based method. 

### 3.3. N-1 Perfusion Rate Controlled by a Capacitance Probe Streamlines Process Development

Development of a tailored N-1 process is necessary for each new recombinant protein due to clone-specific differences such as growth rates, nutrient consumption rates, and waste metabolite production rates. The CSPR for each molecule must be set high enough to support growth to the target cell density yet low enough to not perfuse excess media. The purpose of these experiments was to determine an optimal CSPR for each molecule in development. Specifically, lower CSPR leads to less media perfused during the run; therefore, the goal was to identify the lowest CSPR that supported and maintained a healthy, high cell density culture.

**Figure 4:**
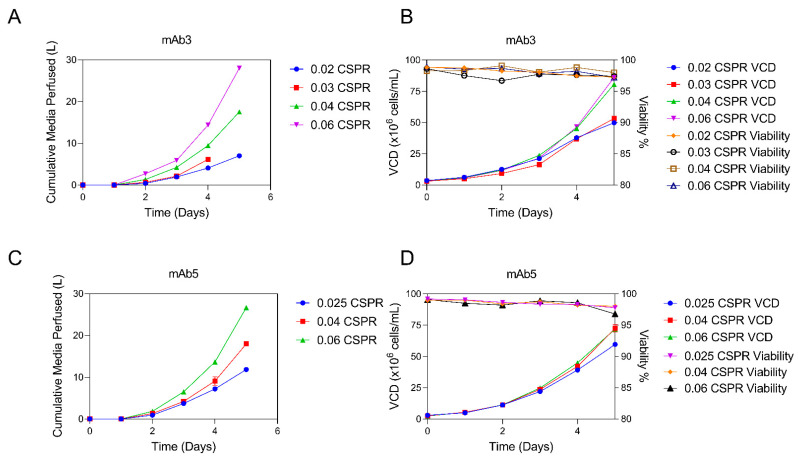
CSPR process development experiment. Comparison of different CSPRs in two mAbs are shown. For mAb3, cumulative media usage for each CSPR tested is plotted in (**A**), and VCD and viability are plotted in (**B**) (n = 1 for each value of CSPR). For mAb5, cumulative media usage for each CSPR tested is plotted in (**C**), and VCD and viability are plotted in (**D**) (n = 1).

The CSPR development experiments for mAb3 and mAb5 are shown in Figure 4. Each experiment tests a range of CSPR between 0.02 and 0.06 nL/cell/day. For both molecules, 0.04 nL/cell/day was selected as the optimal condition. In this experiment, mAb3 had an average doubling time of 26.0 h, and mAb5 had an average doubling time of 24.6 h (Table 1). This perfusion rate led to approximately the same final VCD as the next highest perfusion rate (0.06 nL/cell/day), while using 37% less media for mAb3 and 33% less media for mAb5. The CSPRs tested below 0.04 nL/cell/day exhibited slow growth of the N-1 perfusion cultures, likely due to a lack of nutrients and/or a build-up of inhibitory metabolites; however, neither limiting nor accumulating factors were readily identifiable by standard metabolite analysis for all mAbs (Supplemental Figure S1). Identification of these metabolites may require metabolomic analysis and could be the focus of a future investigation. For mAb3, the 0.03 and 0.02 nL/cell/day CSPR conditions resulted in a 33.9% and 38.2% decrease in final VCD, respectively (Figure 4B), when compared to the final VCD for 0.04 nL/cell/day CSPR. mAb5 showed a 17.7% decrease in final VCD values for 0.025 nL/cell/day CSPR compared to the optimal 0.04 nL/cell/day CSPR (Figure 4D).

The optimized CSPR established during process development for mAb2 through mAb6 are summarized in Table 1. Four out of five mAbs performed well with a CSPR of 0.04 nL/cell/day, regardless of differences in doubling times, inoculation densities, and total process duration (Table 1). The exception was mAb2, which required a higher CSPR of 0.08 nL/cell/day to maintain a high viability, high VCD cell culture. Since most mAbs performed well at a CSPR of 0.04 nL/cell/day, we were able to define that CSPR as a N-1 platform perfusion strategy which could be applied to new molecules coming into upstream process development (mAb1 was developed prior to establishing 0.04 nL/cell/day CSPR as platform and was only tested at 0.03 nL/cell/day). Using 0.04 nL/cell/day CSPR as a platform perfusion rate allows for a platform process that automatically adapts the perfusion rates to cell lines with different doubling times, simplifying the development workflow. 

**Figure 5:**
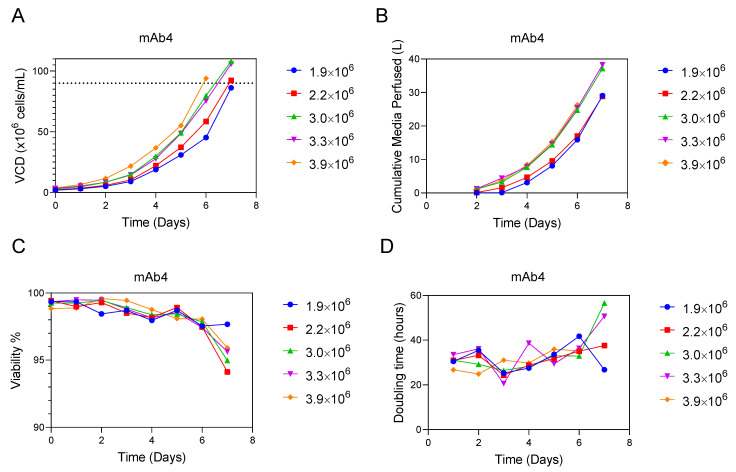
Inoculation VCD process development experiment. Comparison of performance for each inoculation density tested for mAb4 (n = 1 for each inoculation density). VCD (**A**), cumulative media perfused in liters (**B**), viability (**C**), and doubling time (hours) (**D**) are plotted.

Similar to the CSPR experiment, we tested a variety of inoculation densities side-by-side in separate N-1 perfusion bioreactors for mAb4 (Figure 5), using the final VCD required for transfer to the production as the target threshold. While calculating final VCD based on doubling time is a useful starting point, the doubling time increases toward the end of the N-1 (Figure 5D), when VCDs reach high densities, making it difficult to accurately predict the final VCD by extrapolating the VCD based on the doubling time. Furthermore, it is important to confirm that high viability is maintained at the desired final VCD to successfully transfer to the production step. Therefore, an experimental approach to determine the appropriate initial VCD to reach the desired final VCD is necessary. 

N-1 bioreactors were inoculated with VCDs of 1.9 × 10^6^, 2.2 × 10^6^, 3.0 × 10^6^, 3.3 × 10^6^ and 3.9 × 10^6^ cells/mL with the goal of reaching a final N-1 VCD of >90 × 10^6^ cells/mL, which was required to inoculate the production bioreactor at the target initial VCD in the clinical manufacturing facility. Perfusion was initiated one day post inoculation, and CSPR was fixed at 0.04 nL/cell/day for all test conditions and was automatically controlled by the online capacitance measurement. The highest inoculation density of 3.9 × 10^6^ cells/mL reached the target VCD within 6 days, while the inoculation densities ranging from 2.2 × 10^6^ cells/mL to 3.3 × 10^6^ cells/mL reached the target VCD after 7 days (Figure 5A). The lowest inoculation density of 1.9×10^6^ cells/mL failed to reach the target VCD within 7 days.

Inoculating at the higher initial VCD of 3.9 × 10^6^ cells/mL had two advantages over the lower inoculation densities that reached the target final VCD: a shorter duration and decreased media usage. The six-day duration for the 3.9 × 10^6^ cells/mL condition reaching >90 × 10^6^ cells/mL used less media than the seven-day processes for 3.3 × 10^6^ (38% reduction), 3.0 × 10^6^ (30% reduction), and 2.2 × 10^6^ (9% reduction) (Figure 5B). The 3.9 × 10^6^ cells/mL seeding density also maintained high viability (98%) on day 6 (Figure 5C), which is desirable for transfer to the production step. This experiment demonstrates a straightforward determination of the target inoculation VCD that reaches the required final VCD for transfer to production with duration and media usage efficiency. Conversely, if a CSPR had not been used, each different inoculation VCD would have required a different volumetric perfusion strategy, complicating the experimental design.

Thus, a process flow utilizing online capacitance probe control allows for a streamlined development strategy. With just a few (typically two or three) experiments, the optimal CSPR and inoculation density can be identified for a new molecule. In addition, a platform CSPR for this media type was able to be established, providing a good starting point for process development.

### 3.4. Scalability of N-1 Reactors Using Capacitance-Based Perfusion Control

Successful scale-up of the bench scale (5 L) process is required for transfer to manufacturing facilities. To ensure a proper performance of the online controlled perfusion rate at manufacturing scale (500 L), we first assessed performance at the pilot plant scale (200 L). The CSPR of 0.04 nL/cell/day from bench scale optimization for mAbs 4 and 5 was utilized at a larger scale with the same online control setup using biocapcitance probes and the same capacitance probe correction factor determined from experiments at bench scale. 

**Figure 6:**
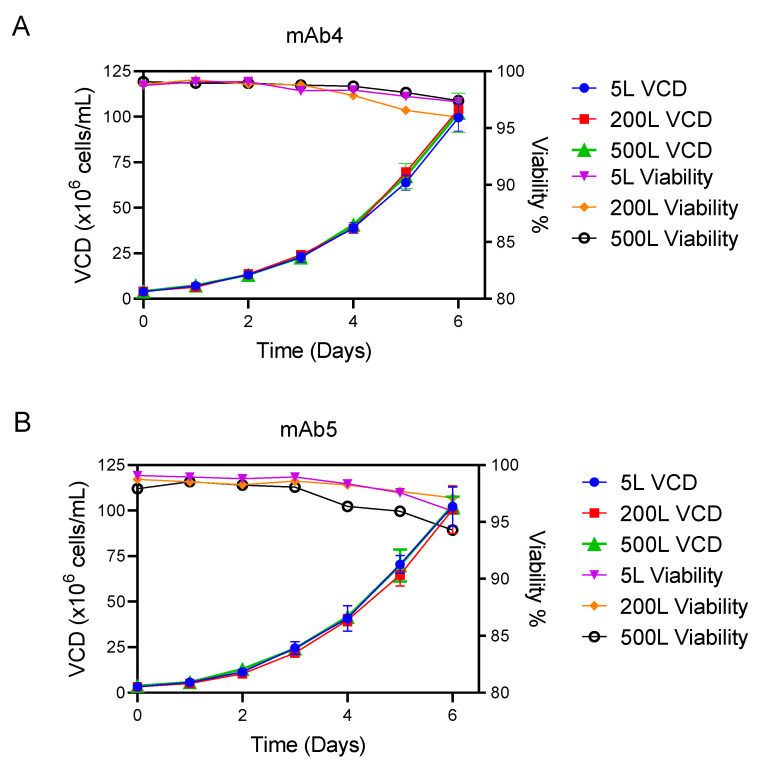
Scale up performance of N-1 perfusion bioreactors controlled with the capacitance probe. Comparison of cell culture performance at different bioreactor scales. VCD and viability are plotted for mAb4 ((**A**); 5 L: n = 4, 200 L: n = 4, 500 L: n = 3) and mAb5 ((**B**); 5 L: n = 5, 200 L: n = 1, 500 L: n = 3). Data are plotted as average ± standard deviation.

The VCD achieved in process scale up to 200 and 500 L (Figure 6) was compared with the process developed at 5 L for two mAbs. For mAb4, the final average VCDs at the 5, 200, and 500 L scales were 100 × 10^6^ cells/mL (n = 5), 104 × 10^6^ cells/mL (n = 1), and 104 × 10^6^ cells/mL (n = 3), respectively (Figure 6A). For mAb5, the final average VCDs at the 5, 200 and 500 L scales were 103 × 10^6^ cells/mL (n = 4), 101 × 10^6^ cells/mL (n = 4), and 103 × 10^6^ cells/mL (n = 3), respectively (Figure 6B). Importantly, the final viability for mAb4 at all scales tested was >96% (Figure 6A) and >92% for mAb4 (Figure 6B), confirming that the seed cultures were suitable for transfer to production bioreactors.

The processes matched well at each scale with similar VCD and metabolite performances across each sampling time point during the entire culture duration (Supplemental Figure S2). We therefore found that establishing the perfusion rate and capacitance probe correction factor at bench scale translated into robust process scalability to pilot plant and manufacturing scales.

### 3.5. Capacitance Optimized N-1 Perfusion Converted to an Optimized Volume-Specific Exchange Rate Process for Facility Fit

The initial driving factor for utilizing an online capacitance probe for automatic perfusion rate control was to optimize media usage. Using a volume-specific exchange rate strategy, it is likely that at any given point in time the perfusion rate will be over-perfusing or under-perfusing media (Figure 2). However, once the online capacitance probe-controlled process has been developed and media usage has been optimized for a particular mAb, an offline volume-specific exchange rate strategy can be derived from the optimized process and used as a backup strategy if the facility encounters a capacitance probe failure during a run. Alternatively, the process can be developed and optimized at bench scale, and then the resulting process can be transferred as an optimized volume-specific exchange rate process to a manufacturing facility that does not have the capability to implement a capacitance probe to control the media perfusion rate. This option will result in less robust control of the process. For example, an online capacitance probe-controlled perfusion rate will automatically account for any minor deviation in inoculation density (e.g., leading to a high or low inoculation density), while an optimized, offline volume-specific exchange rate process will not have this built-in flexibility. However, this conversion to a volume-specific exchange rate perfusion strategy may be required to run the perfusion process if the manufacturing facility does not have the capacitance probe integrated into the control system, or as a backup strategy in the case of a capacitance probe failure.

**Figure 7:**
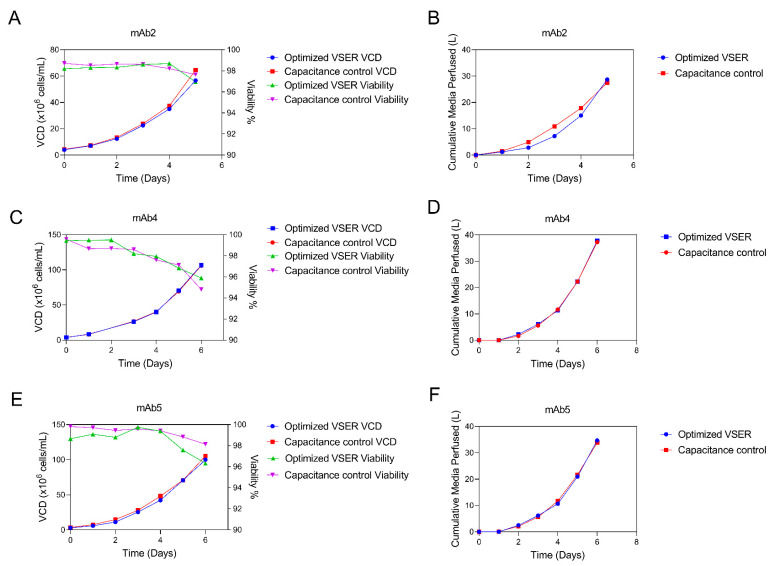
Optimized offline VSER (volume specific exchange rate) based on capacitance developed N-1 process. Comparison of online capacitance probe-controlled perfusion rate versus an optimized, offline volume-specific exchange rate developed based on historic media usage data from online capacitance probe-controlled studies for three mAbs. VCD and viability are plotted for each mAb (**A**,**C**,**E**), and cumulative media perfused for each mAb (**B**,**D**,**F**). n = 1 for each condition.

We demonstrated this optimized, offline volume-specific exchange rate strategy in Figure 7 with mAb2, mAb4, and mAb5. We previously showed the optimization of the N-1 perfusion process in these molecules (Figure 2 and Figure 4) utilizing the perfusion rates controlled by an online capacitance probe. A volume-specific exchange rate based on the online capacitance probe process was calculated for each molecule, and this offline strategy was tested side-by-side for a performance comparison in Figure 7. During development, we observed that mAb4, for example, perfused approximately 0.0 VVD on days 0 to 1, 0.6 VVD on days 1 to 2, 0.9 VVD on days 2 to 3, 1.3 VVD on days 3 to 4, 2.6 VVD on days 4 to 5, and 3.5 VVD on days 5 to 6; thus, this strategy was applied as the optimized, offline volume-specific exchange rate strategy for comparison with the online CSPR-based approach. 

The results of this process comparison revealed a successful offline volume-specific exchange rate process performance. For each mAb tested, the volume-specific exchange rate process performed similarly to the online capacitance probe-controlled process both in terms of media usage and VCD. mAb2 achieved a final VCD of 65 × 10^6^ cell/mL for the online controlled perfusion compared to 57 × 10^6^ cells/mL with the optimized volume-specific exchange rate perfusion, while final cumulative media usage differed by 4%. mAb4 achieved a final VCD of 106 × 10^6^ cells/mL for the online capacitance probe-controlled perfusion compared to 107 × 10^6^ cells/mL with the optimized volume-specific exchange rate perfusion, while final cumulative media usage differed by 1%. mAb5 achieved a final VCD of 105 × 10^6^ cells/mL for the online capacitance probe-controlled perfusion compared to 100 × 10^6^ cells/mL with the optimized volume-specific exchange rate perfusion, while final cumulative media usage differed by 6%. All processes successfully maintained high viability (>95%), which is important for transfer to the production step (Figure 7A,C,E).

## 4. Discussion

In this report, we detail the establishment of an intensified N-1 platform perfusion control strategy using real-time measurements from an in-line capacitance probe. Implementation of this technology was a key enabler of the perfusion platform development discussed in our previous reports [5,6]. Permittivity correlated to offline VCD with high accuracy from relatively low cell densities to high cell densities (greater than 100 × 10^6^ cells/mL) for multiple mAbs (Figure 2), enabling the control of perfusion rate up to those high densities and subsequent inoculation of the production bioreactor at 10 to 20 × 10^6^ cells/mL. This high cell density culture was critical to the significantly increased titers in the new version of our platform, as reported previously [6]. Importantly, the technology was scalable, enabling implementation in a GMP manufacturing environment for mAb4 and mAb5 (Figure 6). While several other reports discuss the development of capacitance technology for control of N-1 perfusion at laboratory scale [3,9], only a few have shown successful scalability and transfer into a manufacturing environment [5,6]. A summary of other perfusion N-1 studies and results are summarized in Table 2 (adapted from [8]).

When compared with two other volumetric platform perfusion control options, the cell-specific automated perfusion control strategy was able to significantly decrease media usage (Figure 3). Efficiency in media usage is another key enabler of implementation of perfusion at large scale, where media volumes are often limited by the size of storage vessels in the large-scale facility [3]. The result found here is indicative of a more general phenomenon: Specifying a volumetric-based perfusion rate a priori often leads to perfusion of more media than required, because the opposite condition (i.e., perfusing less media than required) leads to less healthy cell cultures and thus is generally avoided. While we demonstrated that it is feasible to develop volumetric perfusion control strategies that also exhibited healthy cell growth and efficient media usage (Figure 7), the perfusion rates were derived directly from capacitance probe-controlled development runs. Given the need to accelerate timelines and deliver new medicines to patients quickly, it would not have been practical in a process development setting to arrive at those numbers through trial-and-error experimentation with volume-controlled perfusion rates without the capacitance probe. Thus, if facility fit for implementing capacitance probes is a concern, there is still an advantage for using the probes during development to define a process with efficient media usage.

While the offline optimized volume-specific exchange rate process described in Figure 7 works well, it inherently loses the robust process control and PAT-associated advantages that come with implementing the online capacitance probe in the manufacturing setting. For example, if a deviation in manufacturing leads to the bioreactor being inoculated below or above the targeted range, the capacitance probe-controlled process will automatically adjust for the difference in cells, but the volume-controlled process would not be able to adapt. Furthermore, PAT tools such as the capacitance probe provide more data to scientists when completing root cause investigations, as demonstrated recently by Moore et al. [29]. Due to the advantages afforded by this PAT technology, it is likely that capacitance will be used more often in next-generation manufacturing of biologics.

## 5. Conclusions

In conclusion, we have demonstrated the integration of an in-line capacitance probe for multiple mAbs in an intensified N-1 perfusion platform process. Permittivity was shown to have a strong correlation with VCD up to high cell densities, and the application of in-line capacitance probes has made our N-1 processes more efficient and faster to develop. Media use was reduced by approximately 25% when compared to a platform volume-based perfusion strategy, and the platform development workflow was streamlined by using the CSPR as a single variable that adjusts perfusion rate when the growth rate is shifted (e.g., to account for clone-to-clone differences or differences in process parameters). The technology enabled multiple successful large-scale N-1 perfusion runs including several in a GMP manufacturing setting.

## Figures and Tables

**Table 1:** CSPR platforming study data summary of a representative run at 5 L scale.

	CSPR (nL/Cell/Day)	Glucose in Media (g/L)	Initial VCD (×106 Cells/mL)	Final VCD (×106 Cells/mL)	Duration (Days)	Total Vessel Volumes Perfused	Doubling Time (Hours)
mAb1	0.03	6	1.3	46	6	2.7	27.0
mAb2	0.08	6	3.0	42	5	8.2	42.8
mAb3	0.04	8	3.7	89	5	5.8	26.0
mAb4	0.04	8	3.8	94	6	10.2	30.9
mAb5	0.04	8	2.4	75	5	5.4	24.6
mAb6	0.04	6	1.1	69	6	3.5	27.6

**Table 2:** Summary of perfusion N-1 publications. This table is adapted from [8] with addition of new publication data. NA = not applicable.

Perfusion N-1 Study	Pohlscheidt et al. (2013)	Padawer et al. (2013)	Yang et al. (2014)	Yongky et al. (2019)	Xu et al. (2020)	Xu et al. (2020)	Schulze et al. (2021)	Muller et al. (2022)
Perfusion equipment	Inclined settler	ATF	ATF	Perfusion bioreactor bag	ATF	ATF	Perfusion bioreactor bag	Perfusion bioreactor bag
N-1 bioreactor (L)	3000	3	4	5	500	5, 20, 200	1	1, 2.5, 5, 25
N-1 inoculation VCD (×10^6^ cells/mL)	2	NA	NA	NA	3.74	NA	0.2	0.2
N-1 inoculation cell viability (%)	98	>95	~98	NA	98	98	~98	~98
N-1 culture duration (day)	5	6	5	5	6	6	7	6
Perfusion medium used (L)	4900	18	12	27.5	NA	NA	NA	NA
N-1 final VCD (×10^6^ cells/mL)	13.8	24	40	22–34	103	90–120	100	>100
N-1 final cell viability (%)	90	>95	~97	99	94	>94	96	>95
Subsequent production bioreactor (L)	400	3	4	5	2000	5, 20, 500, 1000	0.015	0.250
Production inoculation VCD from perfusion N-1 (×10^6^ cells/mL)	2	5	10	3–5	16	10–20	0.3	2.5, 5
Production duration from N-1 perfusion (days)	14	8	12	14	14	10–14	7	9, 10
Production inoculation VCD from conventional N-1 (×10^6^ cells/mL)	0.6	0.2	0.4	3–5	3	3–6	0.3	0.3
Production duration from conventional N-1 (days)	16	14	17	14	14	10–14	7	12
Production duration reduction by perfusion N-1 (%)	13	43	29	NA	NA	NA	NA	29, 18
Number of product cell lines tested	1	1	2	3	1	3	1	1

## Data Availability

Not applicable.

## Supplementary Materials

The following supporting information can be downloaded at: https://www.mdpi.com/article/10.3390/bioengineering9040128/s1, Figure S1: Metabolite profiles for CSPR development; Figure S2: Metabolite profiles for scale up bioreactors controlled with the capacitance probe.

## References

[1] Padawer I., Ling W.L.W., Bai Y.L. (2013). Case Study: An accelerated 8-day monoclonal antibody production process based on high seeding densities. Biotechnol. Prog..

[2] Pohlscheidt M., Jacobs M., Wolf S., Thiele J., Jockwer A., Gabelsberger J., Jenzsch M., Tebbe H., Burg J. (2013). Optimizing capacity utilization by large scale 3000 L perfusion in seed train bioreactors. Biotechnol. Prog..

[3] Yang W.C., Lu J., Kwiatkowski C., Yuan H., Kshirsagar R., Ryll T., Huang Y.M. (2014). Perfusion seed cultures improve biopharmaceutical fed-batch production capacity and product quality. Biotechnol. Prog..

[4] Müller D., Klein L., Lemke J., Schulze M., Kruse T., Saballus M., Matuszczyk J., Kampmann M., Zijlstra G. (2022). Process intensification in the biopharma industry: Improving efficiency of protein manufacturing processes from development to production scale using synergistic approaches. Chem. Eng. Process.—Process Intensif..

[5] Xu J., Xu X., Huang C., Angelo J., Oliveira C.L., Xu M., Xu X., Temel D., Ding J., Ghose S. (2020). Biomanufacturing evolution from conventional to intensified processes for productivity improvement: A case study. MAbs.

[6] Xu J.L., Rehmann M.S., Xu M.M., Zheng S., Hill C., He Q., Borys M.C., Li Z.J. (2020). Development of an intensified fed-batch production platform with doubled titers using N-1 perfusion seed for cell culture manufacturing. Bioresour. Bioprocess.

[7] Huang Y.M., Hu W.W., Rustandi E., Chang K., Yusuf-Makagiansar H., Ryll T. (2010). Maximizing Productivity of CHO Cell-Based Fed-Batch Culture Using Chemically Defined Media Conditions and Typical Manufacturing Equipment. Biotechnol. Prog..

[8] Yongky A., Xu J., Tian J., Oliveira C., Zhao J., McFarland K., Borys M.C., Li Z.J. (2019). Process intensification in fed-batch production bioreactors using non-perfusion seed cultures. MAbs.

[9] Schulze M., Lemke J., Pollard D., Wijffels R.H., Matuszczyk J., Martens D.E. (2021). Automation of high CHO cell density seed intensification via online control of the cell specific perfusion rate and its impact on the N-stage inoculum quality. J. Biotechnol..

[10] Xu S., Gavin J., Jiang R.B., Chen H. (2017). Bioreactor Productivity and Media Cost Comparison for Different Intensified Cell Culture Processes. Biotechnol. Prog..

[11] Jordan M., Mac Kinnon N., Monchois V., Stettler M., Broly H. (2018). Intensification of large-scale cell culture processes. Curr. Opin. Chem. Eng..

[12] Woodgate J.M. (2018). Perfusion N-1 Culture-Opportunities for Process Intensification. Biopharm. Process..

[13] Gagnon M., Nagre S., Wang W., Hiller G.W. (2018). Shift to high-intensity, low-volume perfusion cell culture enabling a continuous, integrated bioprocess. Biotechnol. Prog..

[14] Konstantinov K., Goudar C., Ng M., Meneses R., Thrift J., Chuppa S., Matanguihan C., Michaels J., Naveh D. (2006). The “push-to-low” approach for optimization of high-density perfusion cultures of animal cells. Advances in Biochemical Engineering/Biotechnology.

[15] Dowd J.E., Jubb A., Kwok K.E., Piret J.M. (2003). Optimization and control of perfusion cultures using a viable cell probe and cell specific perfusion rates. Cytotechnology.

[16] Wasalathanthri D.P., Rehmann M.S., Song Y., Gu Y., Mi L., Shao C., Chemmalil L., Lee J., Ghose S., Borys M.C. (2020). Technology outlook for real-time quality attribute and process parameter monitoring in biopharmaceutical development-A review. Biotechnol. Bioeng..

[17] Chong L., Saghafi M., Knappe C., Steigmiller S., Matanguihan C., Goudar C.T. (2013). Robust on-line sampling and analysis during long-term perfusion cultivation of mammalian cells. J. Biotechnol..

[18] Derfus G.E., Abramzon D., Tung M., Chang D., Kiss R., Amanullah A. (2010). Cell culture monitoring via an auto-sampler and an integrated multi-functional off-line analyzer. Biotechnol. Prog..

[19] Lee H.W., Carvell J., Brorson K., Yoon S. (2015). Dielectric spectroscopy-based estimation of VCD in CHO cell culture. J. Chem. Technol. Biot..

[20] Lu F., Toh P.C., Burnett I., Li F., Hudson T., Amanullah A., Li J.C. (2013). Automated dynamic fed-batch process and media optimization for high productivity cell culture process development. Biotechnol. Bioeng..

[21] Metze S., Ruhl S., Greller G., Grimm C., Scholz J. (2020). Monitoring online biomass with a capacitance sensor during scale-up of industrially relevant CHO cell culture fed-batch processes in single-use bioreactors. Bioprocess Biosyst. Eng..

[22] Morris C., Madhavarao C.N., Yoon S., Ashraf M. (2021). Single in-line biomass probe detects CHO cell growth by capacitance and bacterial contamination by conductivity in bioreactor. Biotechnol. J..

[23] Berry B., Moretto J., Matthews T., Smelko J., Wiltberger K. (2015). Cross-Scale Predictive Modeling of CHO Cell Culture Growth and Metabolites Using Raman Spectroscopy and Multivariate Analysis. Biotechnol. Prog..

[24] Chen G., Hu J., Qin Y.J., Zhou W.C. (2021). Viable cell density on-line auto-control in perfusion cell culture aided by in-situ Raman spectroscopy. Biochem. Eng. J..

[25] Li M.Y., Ebel B., Chauchard F., Guedon E., Marc A. (2018). Parallel comparison of in situ Raman and NIR spectroscopies to simultaneously measure multiple variables toward real-time monitoring of CHO cell bioreactor cultures. Biochem. Eng. J..

[26] Whelan J., Craven S., Glennon B. (2012). In situ Raman spectroscopy for simultaneous monitoring of multiple process parameters in mammalian cell culture bioreactors. Biotechnol. Prog..

[27] Dabros M., Dennewald D., Currie D.J., Lee M.H., Todd R.W., Marison I.W., von Stockar U. (2009). Cole-Cole, linear and multivariate modeling of capacitance data for on-line monitoring of biomass. Bioprocess Biosyst. Eng..

[28] Olsson L., Nielsen J. (1997). On-line and in situ monitoring of biomass in submerged cultivations. Trends Biotechnol..

[29] Moore B., Sanford R., Zhang A. (2019). Case study: The characterization and implementation of dielectric spectroscopy (biocapacitance) for process control in a commercial GMP CHO manufacturing process. Biotechnol. Prog..

[30] Zhang A., Tsang V.L., Moore B., Shen V., Huang Y.M., Kshirsagar R., Ryll T. (2015). Advanced process monitoring and feedback control to enhance cell culture process production and robustness. Biotechnol. Bioeng..

[31] Tian J., He Q., Oliveira C., Qian Y., Egan S., Xu J., Qian N.X., Langsdorf E., Warrack B., Aranibar N. (2020). Increased MSX level improves biological productivity and production stability in multiple recombinant GS CHO cell lines. Eng. Life Sci..

[32] Su Y.N., Wei Z.H., Miao Y.N., Sun L.L., Shen Y.N., Tang Z.R., Li L., Quan Y.F., Yu H.Y., Wang W.C. (2021). Optimized process operations reduce product retention and column clogging in ATF-based perfusion cell cultures. Appl. Microbiol. Biot..

[33] Stepper L., Filser F.A., Fischer S., Schaub J., Gorr I., Voges R. (2020). Pre-stage perfusion and ultra-high seeding cell density in CHO fed-batch culture: A case study for process intensification guided by systems biotechnology. Bioprocess Biosyst. Eng..

