# Supplementary material for: N-1 Perfusion Platform Development Using a Capacitance Probe for Biomanufacturing
Source: Bioengineering (Basel). 2022 Mar 22;9(4):128. doi: 10.3390/bioengineering9040128 (PMC9029935; doi:10.3390/bioengineering9040128)
Supplement: Supplementary file 1 [file bioengineering-09-00128-s001.zip › bioengineering-1537840-supplementary.pdf]

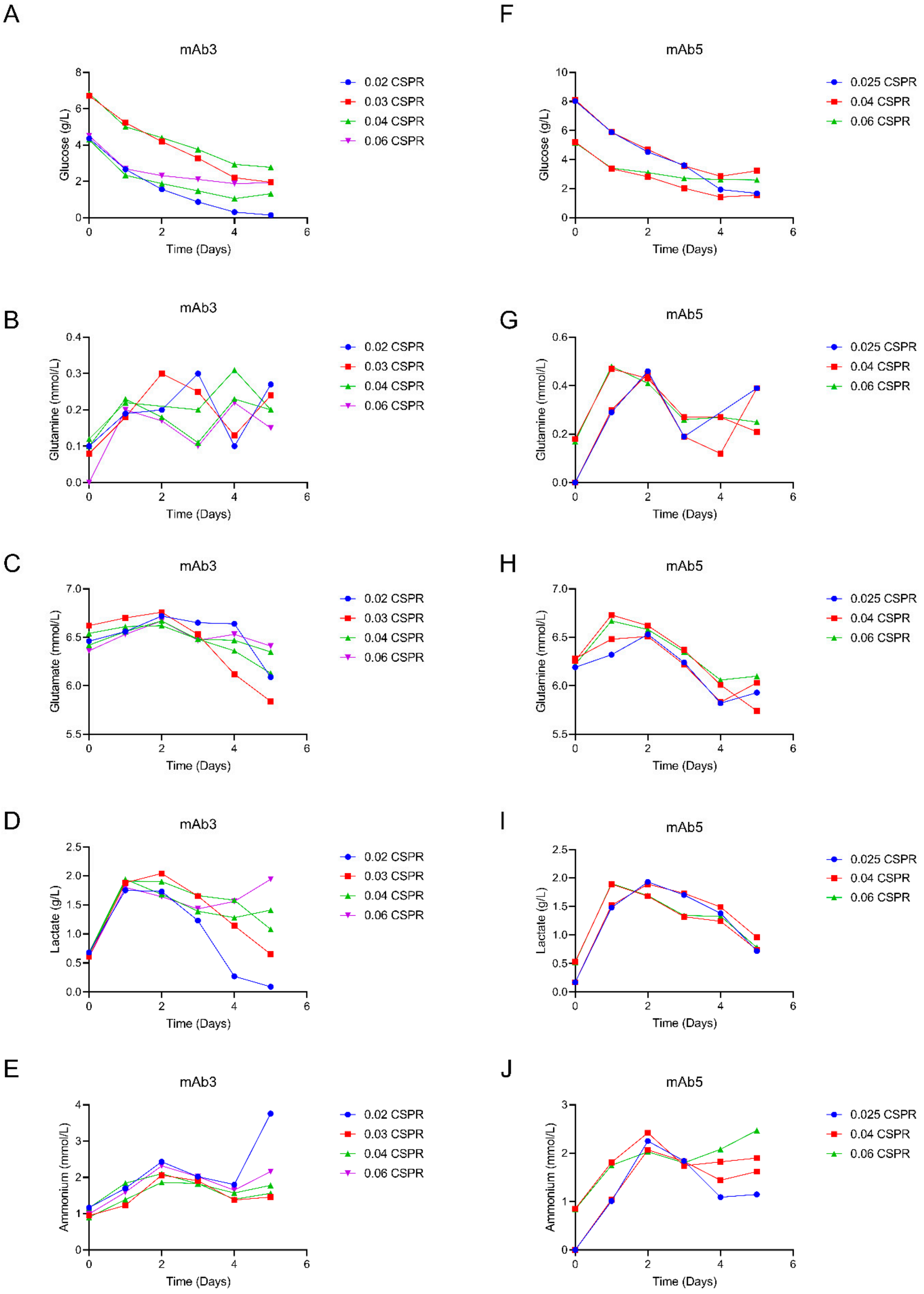
A
mAb3
Glucose (g/L)
Time (Days)
0.02 CSPR
0.03 CSPR
0.04 CSPR
0.06 CSPR
B
mAb3
Glutamine (mmol/L)
Time (Days)
0.02 CSPR
0.03 CSPR
0.04 CSPR
0.06 CSPR
C
mAb3
Glutamate (mmol/L)
Time (Days)
0.02 CSPR
0.03 CSPR
0.04 CSPR
0.06 CSPR
D
mAb3
Lactate (g/L)
Time (Days)
0.02 CSPR
0.03 CSPR
0.04 CSPR
0.06 CSPR
E
mAb3
Ammonium (mmol/L)
Time (Days)
0.02 CSPR
0.03 CSPR
0.04 CSPR
0.06 CSPR
F
mAb5
Glucose (g/L)
Time (Days)
0.025 CSPR
0.04 CSPR
0.06 CSPR
G
mAb5
Glutamine (mmol/L)
Time (Days)
0.025 CSPR
0.04 CSPR
0.06 CSPR
H
mAb5
Glutamine (mmol/L)
Time (Days)
0.025 CSPR
0.04 CSPR
0.06 CSPR
I
mAb5
Lactate (g/L)
Time (Days)
0.025 CSPR
0.04 CSPR
0.06 CSPR
J
mAb5
Ammonium (mmol/L)
Time (Days)
0.025 CSPR
0.04 CSPR
0.06 CSPR

1

Supplemental figure 1. Metabolite profiles for CSPR development. For mAb3, glucose, glutamine, glutamate, lactate, ammonium are plotted for each CSPR tested in A, B, C, D, E, respectively. For mAb5, glucose, glutamine, glutamate, lactate, ammonium are plotted for each CSPR tested in F, G, H, I, J. Each line plotted represents one bioreactor (n=1). Metabolites were measured using Roche Cedex.

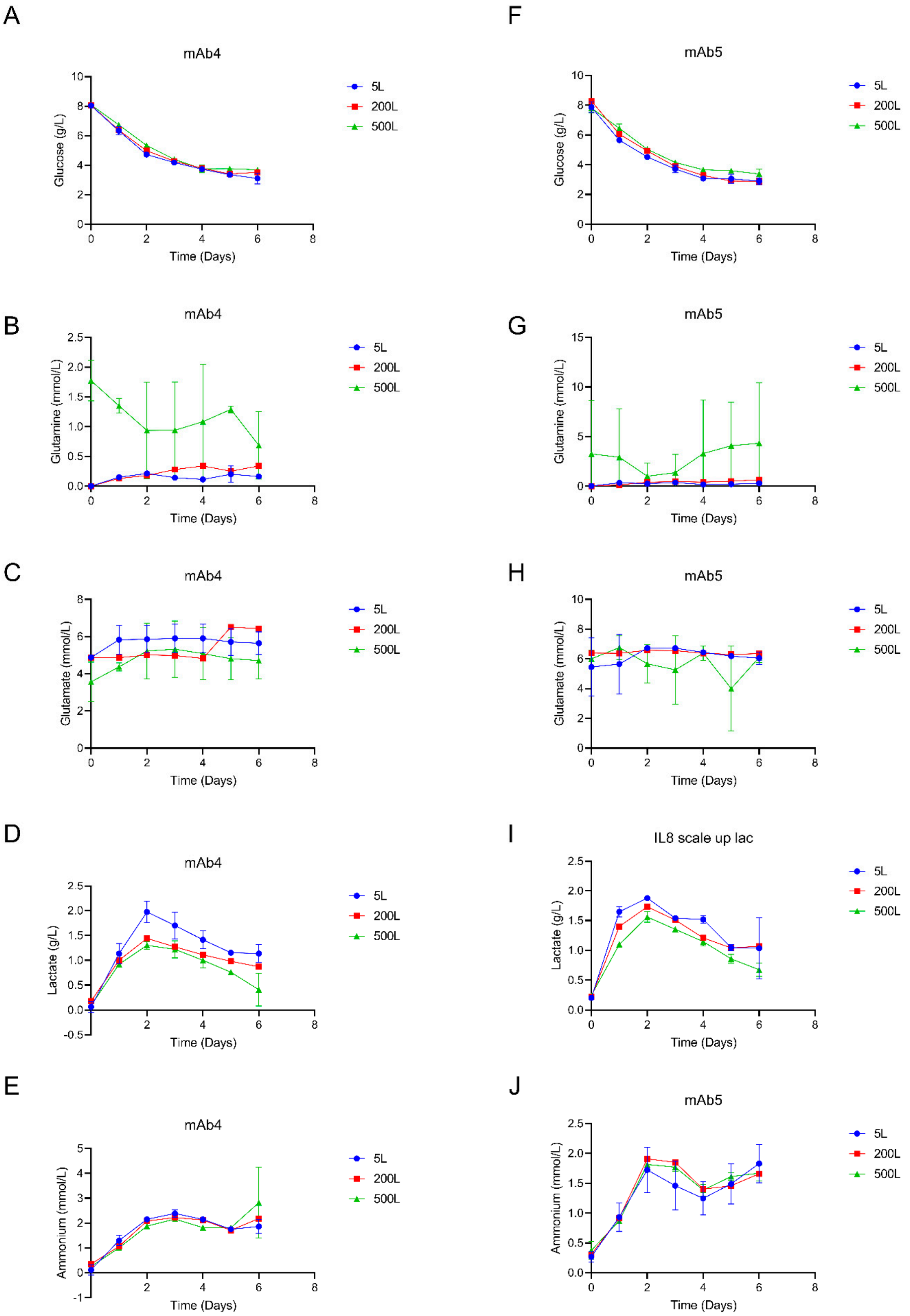
A
mAb4
Glucose (g/L)
Time (Days)
5L
200L
500L
F
mAb5
Glucose (g/L)
Time (Days)
5L
200L
500L
B
mAb4
Glutamine (mmol/L)
Time (Days)
5L
200L
500L
G
mAb5
Glutamine (mmol/L)
Time (Days)
5L
200L
500L
C
mAb4
Glutamate (mmol/L)
Time (Days)
5L
200L
500L
H
mAb5
Glutamate (mmol/L)
Time (Days)
5L
200L
500L
D
mAb4
Lactate (g/L)
Time (Days)
5L
200L
500L
I
IL8 scale up lac
Lactate (g/L)
Time (Days)
5L
200L
500L
E
mAb4
Ammonium (mmol/L)
Time (Days)
5L
200L
500L
J
mAb5
Ammonium (mmol/L)
Time (Days)
5L
200L
500L

Supplemental figure 2. Metabolite profiles for scale up bioreactors controlled with the capacitance probe. For mAb4, glucose, glutamine, glutamate, lactate, ammonium are plotted for each CSPR tested in A, B, C, D, E, respectively. For mAb5, glucose, glutamine, glutamate, lactate, ammonium are plotted for each CSPR tested in F, G, H, I, J. For 5L n=3, for 200L, n=1, 500L n=3. 5L and 200L metabolites were measured using Roche Cedex and 500L metabolites were measured using Nova BioProfile FLEX2.
